# System Dynamics Modeling of Scale Formation in Membrane Distillation Systems for Seawater and RO Brine Treatment

**DOI:** 10.3390/membranes14120252

**Published:** 2024-11-28

**Authors:** Yonghyun Shin, Jaewuk Koo, Sangho Lee

**Affiliations:** 1 Korea Institute of Civil Engineering and Building Technology, 283 Goyangdar-Ro, Ilsan-Gu, Goyang-Si 411-712, Republic of Korea; shinyonghyun@kict.re.kr (Y.S.); koojaewuk@kict.re.kr (J.K.); 2 Civil and Environmental Engineering, Kookmin University, 77 Jeongneung-ro, Seongbuk-gu, Seoul 136-702, Republic of Korea; 3 Water Technologies Innovation Institute and Research Advancement (WTIIRA), Saudi Water Authority (SWA), Al-Jubail 31951, Saudi Arabia

**Keywords:** membrane distillation, scaling, optical coherence tomography, system dynamics

## Abstract

To overcome the limitations of traditional Reverse Osmosis (RO) desalination, Membrane Distillation (MD) has gained attention as an effective solution for improving the treatment of seawater and RO brine. Despite its potential, the formation of inorganic scales, particularly calcium sulfate (CaSO_4_), continues to pose a major challenge. This research aims to explore the scaling mechanisms in MD systems through a combination of experimental analysis and dynamic modeling. Using real seawater and RO brine as feed sources, the scaling behavior was examined under various operational conditions, such as temperature and feed concentration. Optical Coherence Tomography (OCT) was utilized to monitor the real-time development of fouling layers, offering valuable insights into surface crystal formation processes. A System Dynamics Model (SDM) was created based on the experimental data to predict flux decline trends with precision. The model correlated well with experimental observations, highlighting key factors that drive scaling severity. This integrated approach deepens our understanding of scaling dynamics and provides actionable strategies to mitigate fouling in MD systems, thereby enhancing the efficiency and stability of MD desalination operations. Ultimately, this study underscores the potential of combining OCT with system dynamics modeling as a powerful approach for visualizing and validating scaling processes, offering a practical framework for optimizing MD performance and contributing to more sustainable desalination practices.

## 1. Introduction

The scarcity of water has become a pressing concern due to the imbalance between water demand and available resources, driving the rapid growth of seawater desalination technologies over the past two decades [1,2,3]. Although Reverse Osmosis (RO) remains the primary method for seawater desalination [4,5,6,7], it faces challenges such as the environmental impact of brine discharge and high energy consumption. Recently, MD has received increasing attention as a promising alternative that can address these limitations [8,9,10]. Combining RO and MD helps reduce the brine volume and energy use, providing a more sustainable and efficient solution for seawater desalination [11,12].

However, membrane fouling due to the deposition of inorganic scales is a critical issue in MD applications [13]. Typical scaling agents such as calcium sulfate, calcium carbonate, and silica (SiO_2_) precipitate onto the membrane surface, affecting its performance [14,15]. The scale formation process is complex, involving surface (heterogeneous) and bulk (homogeneous) crystallization mechanisms [13,14,16]. Concentration polarization further accelerates scaling by enhancing surface crystal formation [14,17,18], and other factors, including pH [19], temperature [20], and metal ion species [21,22] can also influence this process.

In VMD (Vacuum MD), the primary operating conditions that impact scaling and flux include membrane characteristics, the feedwater temperature, and permeate vacuum pressure. In this study, a commonly used, commercially available PVDF (Polyvinylidene fluoride) membrane was selected for consistency, and the vacuum pressure was set to an optimal level of 0.15 bar to minimize membrane wetting while enhancing the flux. Although increasing the vacuum level can improve the flux and accelerate scale formation, this optimal pressure was maintained to mitigate the risk of wetting [23]. As the feedwater type and temperature are also critical variables in the analysis, these conditions were systematically varied to assess their impact on scaling.

One of the methods that have potential to provide comprehensive information on MD scaling is OCT, which is a non-invasive technique using low-coherence light to perform high-resolution cross-sectional imaging. This technology is widely used in medical fields, allowing for the detailed visualization of microstructural layers. Recent studies highlight OCT’s expanding applications in engineering applications including membrane processes [24]. This is because OCT enables the real-time monitoring of fouling on membrane surfaces without the need of an autopsy [25]. OCT has been adapted for in situ observations of scaling in MD systems, offering insights into the effects of operational parameters on fouling behavior [26,27].

System dynamics (SD) is another approach used to aid the understanding of scale formation phenomena in MD processes. An SD model provides a framework for analyzing complex, dynamic systems by simulating their interrelated components and feedback loops over time [28]. This modeling approach, which is widely applicable across fields such as economics, environmental science, and public health, visualizes potential future scenarios based on current trends and interventions [29]. SD provides a holistic view of the magnitude of complex dynamics, feedback processes, and interdependencies [30]. Nevertheless, few studies have reported the application of SD to interpret membrane fouling and scale formation.

This study investigates CaSO_4_ scaling in MD using experimental data and dynamic modeling. Real seawater and RO brine were treated to analyze scaling under a range of conditions, and a dynamic model was established to predict flux decline accurately. The model, verified using experimental data, identified key parameters affecting scaling and was supported by OCT observations, which provided a deeper understanding of scaling pathways and model refinement. This integrated approach offers strategies for mitigating scaling in MD systems and enhancing operational efficiency.

## 2. Materials and Methods

### 2.1. Laboratory VMD System

**Figure 1:**
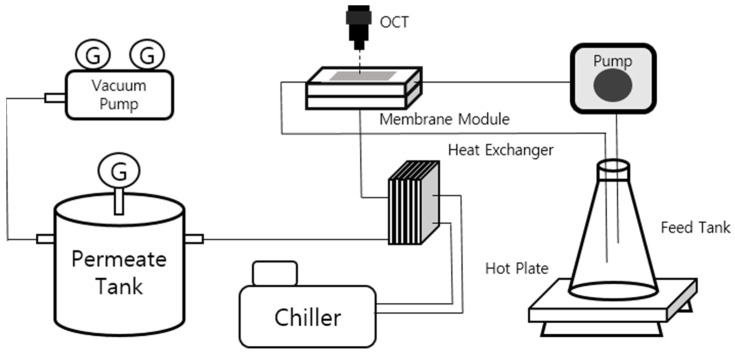
Schematic diagram of VMD system.

A lab-scale VMD system was established to evaluate water flux and fouling behavior, as depicted in Figure 1. Membranes made of polyvinylidene fluoride (Millipore, Burlington, MA, USA) were used in this study. The experimental setup comprised a feed tank, a heater, and a gear pump to circulate the heated feed water, as well as a vacuum pump, a permeate tank, and a chiller for condensing the produced vapor. The vacuum pump was utilized to maintain reduced pressure from the permeate tank through to the downstream side of the membrane, enabling efficient vapor transport across the membrane. For permeate condensation, a plate heat exchanger connected to a chiller was used to ensure effective cooling. To compensate for the thermal energy lost during vapor production, the feed water was heated continuously via a hot plate.

Water flux was monitored using a balance connected with a PC, while conductivity measurements were recorded every 30 min to assess membrane wettability and scaling in the feed tank. The feed temperature was controlled at 60 °C and 70 °C, with a feed flow rate of 0.6 L/min, and the vacuum pressure was set to 0.15 bar. The hydrophobic flat sheet membranes employed in the experiments had an average pore size of 0.22 μm, a thickness of 125 μm, and a porosity of 75%.

### 2.2. Analysis of Membrane Surface

#### 2.2.1. Optical Coherence Tomography (OCT)

OCT was utilized to obtain real-time images of foulants on the membrane surface during the MD experiments. The system featured a source laser (Axsun Tech Pittsburgh, PA, USA) with a center wavelength of 1310 nm and a 100 nm tuning range, enabling the precise visualization of fouling layer development. The OCT setup included a collimator, a galvanometer scanner, and an objective lens to guide and collect light from the membrane surface. Light interference data were processed using Mach-Gen-Loader interferometry(Thorlabs, Newton, NJ, USA), and the resulting optical signals were converted to electrical signals via a balanced amplification photodetector (PDB450C Company, Thorlabs, Newton, NJ, USA).

**Figure 2:**
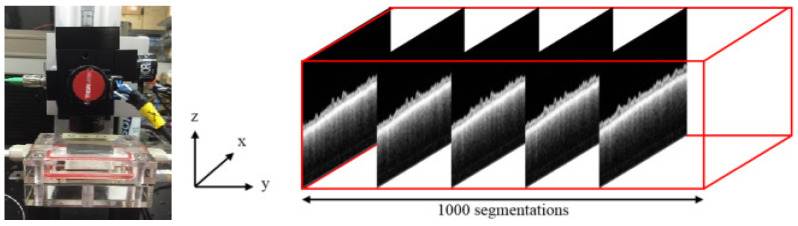
The apparatus of optical coherence tomography.

These signals were digitized using a high-speed digitizer (ATS9350, AlazarTech Inc., Pointe-Claire, QC, Canada), allowing for the generation of intensity-based images that displayed the membrane’s fouling in real time (see Figure 2). The non-invasive nature of OCT enabled continuous monitoring without disrupting the filtration process, offering valuable insights into the growth and structure of fouling layers over time. The resulting OCT images provided detailed information on the fouling layers’ thickness and morphology, which is critical for evaluating membrane performance.

OCT has proven to be highly effective for membrane fouling research. For example, Yang et al. (2020) successfully applied OCT to examine fouling and scaling mechanisms, highlighting its value in providing real-time, high-resolution imaging for membrane surface analysis [31]. Additionally, OCT enables the non-invasive, in situ monitoring of biofilm growth, scaling, and other fouling types in membrane systems, providing both 2D and 3D visualizations of structural characteristics, which are critical for understanding the spatial distribution and dynamics of fouling layers. This capability allows researchers to evaluate cleaning strategies effectively and optimize membrane operations based on real-time data [29,30].

#### 2.2.2. Scanning Electron Microscopy (SEM) with Energy-Dispersive X-Ray Spectroscopy (EDX)

A SEM-EDX (JSM-7160F, JEOL, Tokyo, Japan) was employed to analyze the molecular structures and elemental composition of the fouling on the membrane surface. Before imaging, the MD membranes were dried and coated with platinum to enhance conductivity. SEM provided high-resolution images, enabling the detailed observation of the fouling layers, while EDX facilitated the identification of specific elements within the fouling deposits. This combination allowed for a comprehensive characterization of the membrane surface contamination.

### 2.3. Feed Solution

The feed water used in this study included raw seawater and seawater Reverse Osmosis (SWRO) brine. The seawater was sourced from Gijang-gun, Korea, via The Institute of Fisheries Sciences at Pukyong National University, while the RO brine was collected from an RO plant located in Gwangyang-gun. The concentrations of total dissolved solids (TDS) were around 35,000 mg/L for seawater and 54,400 mg/L for the RO brine, reflecting their differing salinity levels. Prior to the Membrane Distillation (MD) experiments, the seawater underwent a pre-treatment step using a GF/C filter to remove large particles and impurities. The detailed composition of both feed waters is provided in Table 1, offering a comprehensive overview of the chemical characteristics and elemental components used in the experiments.

## 3. Model Development

### 3.1. Mechanisms for the Scale Formation

**Figure 3:**
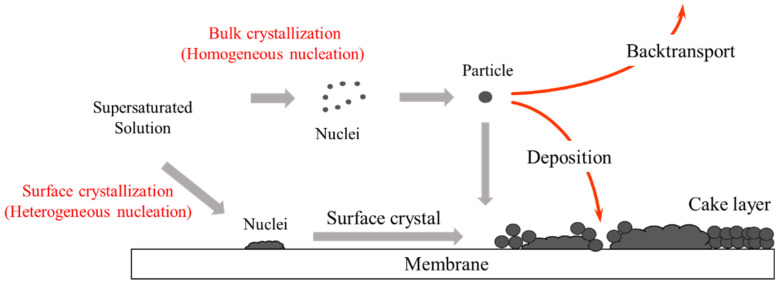
Proposed mechanisms of scale formation in MD systems.

Understanding MD scale formation requires an in-depth examination of both crystallization behaviors and hydrodynamic factors. According to the literature [13,14,17], two distinct crystallization pathways are primarily responsible for scale formation: homogeneous bulk crystal formation and heterogeneous surface crystal formation. Bulk crystal formation occurs when crystals form within the bulk solution and subsequently deposit onto the membrane, leading to the development of cake-like layers that contribute to increased hydraulic resistance. On the other hand, surface crystal formation is characterized by the direct growth of crystals on the membrane surface itself, which obstructs the pores and significantly reduces the active membrane area. Figure 3 visually represents these two distinct crystallization pathways in MD systems.

To analyze the fouling phenomena caused by scaling under different operational conditions, a resistance-in-series model was employed, incorporating elements from both concentration polarization theory and crystallization kinetics [18]. In contrast to previous approaches, this model was designed to account for both the bulk and surface crystal formation processes, providing a more comprehensive understanding of scaling behavior. Consequently, the flux equation was derived to predict performance decline with greater accuracy.

(1) $$J_{v}=B_{m}\Delta P_{v}=\frac{\Delta P_{v}}{\frac{1}{B_{m,0}}+\eta R_{c}}\times \frac{A-A_{b}}{A}$$

In this equation, $B_{m}$ refers to the vapor transport parameter, $∆P_{v}$ represents the vapor pressure difference, η indicates the viscosity of the permeate, $R_{c}$ signifies the resistance attributed to cake formation, A corresponds to the total membrane area, and $A_{b}$ denotes the portion of the membrane area occupied by surface crystals. It should be noted that there is a fundamental difference in the scale formation model between the RO and MD, which is attributed to the different driving forces between them. Accordingly, there is no need to consider osmotic pressure in the current model for MD.

As the thickness of the crystal layer may be assumed to be constant [22], $A_{b}$ can be formulated as follows:

(2) $$A_{b}=\beta m_{s}$$

In this context, β denotes the area covered per unit mass, while $m_{s}$ refers to the mass of the scale that has formed directly on the membrane surface. By assuming that the crystal layers are neither compressible nor deformable, the cake resistance $R_{c}$ can be calculated using the Darcy’s law [18]:

(3) $$R_{c}=\frac{\alpha m_{c}}{A}$$

In this formula, α indicates the specific resistance of the cake layer, while $m_{c}$ stands for the total mass of the precipitated scale that has accumulated.

### 3.2. Estimation of Wall Concentration

Concentration polarization leads to a disparity between $c_{w}$ and $c_{b}$ (the bulk solute concentration). By integrating the film model theory with Fick’s law of diffusion, concentration polarization can be expressed as follows:

(4) $$\frac{c_{w}-c_{p}}{c_{b}-c_{p}}\approx \frac{c_{w}}{c_{b}}=\theta =e^{\frac{J_{v}}{k}}$$

In this expression, k denotes the mass transfer coefficient governing the back diffusion of solutes from the surface to the bulk phase on the feed side, while θ refers to the concentration polarization modulus. For crossflow membrane systems, the mass transfer coefficients are defined as follows [28]:

(5) $$k=1.86\times ({\frac{uD^{2}}{d_{h}L})}^{0.33}\;\;:\mathrm{laminar}\;\mathrm{flow}$$

(6) $$k=0.023\times \left(\frac{u^{0.8}D^{0.67}}{d_{h}^{0.2}\nu^{0.47}}\right)\;:\mathrm{turbulent}\;\mathrm{flow}$$

In this formula, k refers to the mass transport coefficient, u represents the crossflow velocity, D stands for the diffusion constant of salts, $d_{h}$ corresponds to the hydraulic diameter of the flow channel, L indicates the effective length of the module, and *ν* represents the kinematic viscosity of the feed solution. By integrating Equation (4) with either Equation (5) or Equation (6), $c_{w}$ can be calculated from $c_{b}$.

### 3.3. Induction Time and Crystal Growth Rate

The induction time, which is the time needed for detectable crystals to develop, is a critical factor influencing scaling on MD membranes. If the induction time is considered to be proportional to the nucleation rate, it can be represented as *τ*:

(7) $$\tau =\tau_{0}e^{\left(\frac{C\sigma^{3}}{T^{3}\ln(Sw{)}^{2}}\right)}$$

In this equation, τ is a constant linked to the frequency factor, C indicates a constant that characterizes physical properties, *σ* represents the crystal surface energy, T stands for the absolute temperature, and *S_w_* is the supersaturation ratio (=[Ca^2+^][SO_4_^2−^]/$K_{sp}$) [18,32]. The $K_{sp}$ of CaSO_4_ was calculated using the following empirical equation from ASTM D 4692-01 and the thesis by G. Azimi [33]:

(8) $$K_{sp}=0.0001\left(1.1802I_{s}-6.6149{I_{s}}^{2}+22.163{I_{s}}^{3}+0.6981\right)\;\times \left(-7\times 10^{-5}T^{2}+6.17\times 10^{-3}T+0.87\right)$$

where $I_{s}$ is the ionic strength. The rate at which surface crystals grow for scale-forming salts can be represented as follows:

(9) $$\frac{dm_{s}}{dt}=k_{s}(A-A_{b})(c_{w}-c_{s}{)}^{n}=k_{s}A(1-\frac{\beta m_{s}}{A})(\theta c_{b}-c_{s}{)}^{n}$$

In this equation, $k_{s}$ indicates the rate constant for surface crystal formation, $c_{s}$ refers to the saturation concentration, and *n* denotes the reaction order. On the other hand, if bulk crystal formation occurs on the surface of suspended crystal particles, the mass of the resulting cake crystals can be expressed as:

(10) $$\frac{dm_{c}}{dt}=k_{c}s_{p}\psi (c_{b}-c_{s}{)}^{m}=k_{b}(c_{b}-c_{s}{)}^{m}$$

In this equation, $k_{c}$ represents the rate constant for bulk crystal formation, $s_{p}$ corresponds to the surface area of active sites on the bulk crystals, $c_{b}$ indicates the bulk phase concentration, and ψ denotes the deposition probability of crystal particles. Additionally, m is the reaction order, and kc also functions as the apparent rate constant for bulk crystal formation ($k_{c}s_{p}\psi$).

The primary difference between Equations (9) and (10) lies in the crystallization driving force. For surface crystal formation, the driving force is determined by the concentration difference between $c_{w}$ and $c_{s}$, whereas for bulk crystal formation, it is defined by ($c_{b}$ − $c_{s}$). This implies that concentration polarization significantly influences surface crystal formation only. Furthermore, there may be a difference in the induction time for the formation of surface and bulk crystals.

### 3.4. Solution Method

In a batch-operated MD process, J, $c_{b}$, and $c_{p}$ are variable because the concentrate volume ($V_{c}$) changes continuously. The time-dependent variations of $c_{b}$ and $V_{c}$ for a membrane with surface area A can be represented as follows:

(11) $$\frac{d\left(c_{b}V_{c}\right)}{dt}=-\frac{dm_{c}}{dt}-\frac{dm_{s}}{dt}$$

(12) $$\frac{dV_{c}}{dt}=-J_{v}A$$

**Figure 4:**
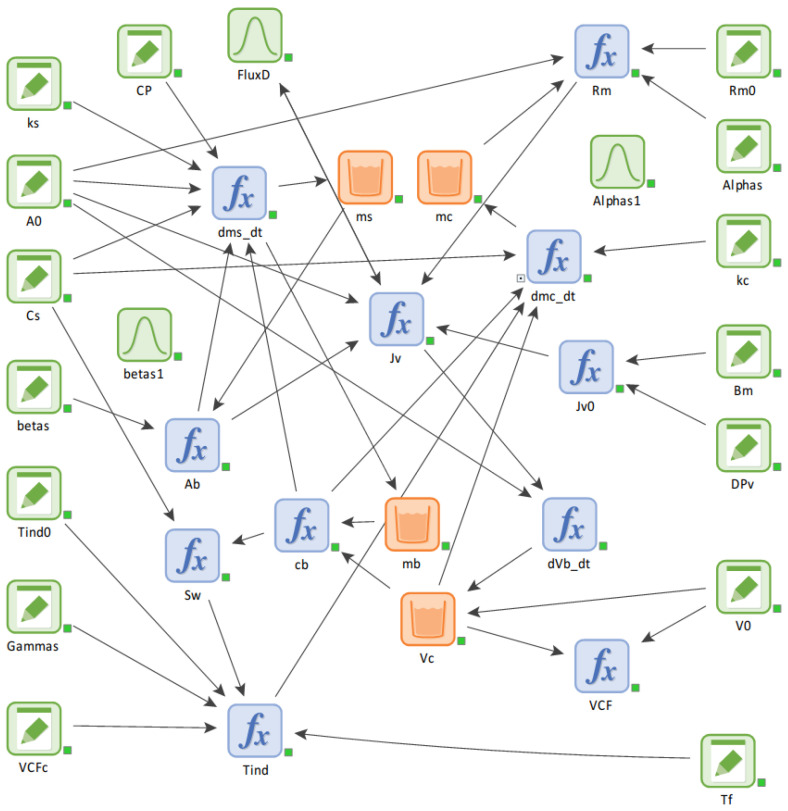
System dynamics model for analysis of scale formation in MD system.

*A_t_* = 0, $c_{b}$ = $c_{f}$ and $V_{c}$ = $V_{f}$, where $c_{f}$ indicates the initial solute concentration and $V_{f}$ is the initial feed volume. The volume concentration factor (VCF) is calculated as the ratio of $V_{f}$ to $V_{c}$. A summary of all parameters used in this study can be found in Table 2, while the procedure for solving the model equations is depicted in Figure 3. The values for *α*, *β*, $k_{s}$ and $k_{b}$ were set based on a previous work on CaSO_4_ scale formation [18]. Then, a system dynamics model was developed using the GoldSim software 14.0 (GoldSim Technology Group LLC, Seattle, WA, USA) (Figure 4). The correlations among key variables such as $m_{b}$, $m_{c}$, $m_{s}$, and $V_{c}$ were simultaneously considered.

### 3.5. Procedures of System Dynamics Modeling

A system dynamics model was constructed based on GoldSim software, which simulates the intricate interactions leading to scaling on membrane surfaces, using the model equations. In contrast to the traditional mathematical models, the SD models offer several advantages. First, SD models are characterized by the complete exposure of all elements and functional relationships, allowing the elucidation of the relationships among elements. Second, SD models are object-oriented, thereby facilitating their expansion and scale-up. Moreover, both deterministic and probabilistic simulations can be incorporated into SD models. In this context, the SD model in the present study differs from the authors’ previous mathematical model.

**Figure 5:**
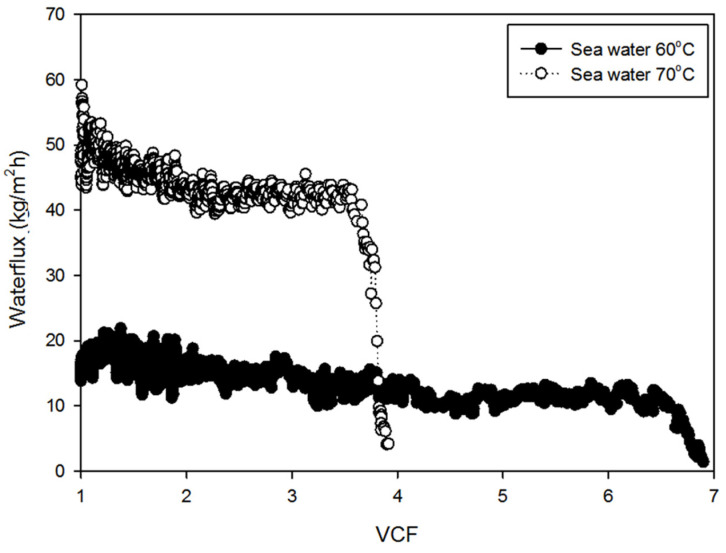
Waterflux by seawater operation: 60 °C and 70 °C.

A causal loop diagram was constructed by reflecting the feedback loop structures associated with the MD scale-formation process. The SD model uses built-in elements provided by GoldSim for entering data and manipulating variables. The data elements, indicated by green pencil symbols, contain the input parameters and constants utilized by the model. The reservoir elements (orange) and the expression elements (blue) correspond to variables and mathematical equations, respectively. The arrows indicate an influence, which visualizes the dependency of one element on another. The fluences were automatically created based on the equations in the expression elements. The overall procedure for solving the SD model is depicted in Figure 5.

## 4. Results and Discussion

### 4.1. Experimental Results and Flux Decline Analysis

**Figure 6:**
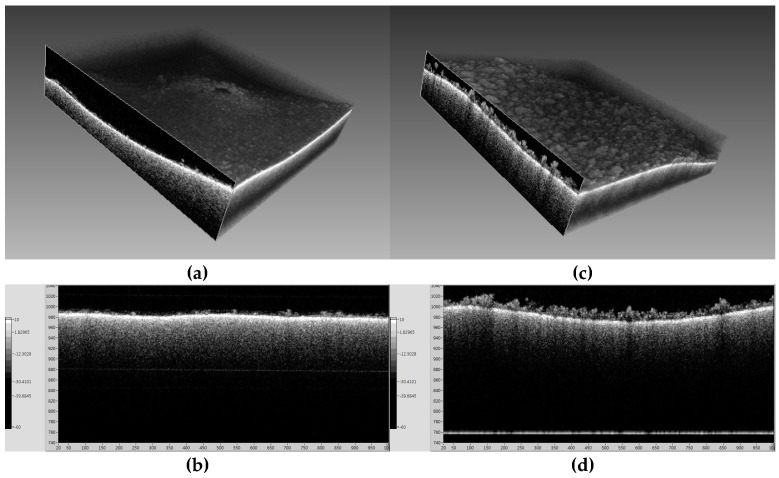
OCT images of membrane surface after (**a**,**b**) 60 °C and (**b**,**d**) 70 °C operation by seawater.

**Figure 7:**
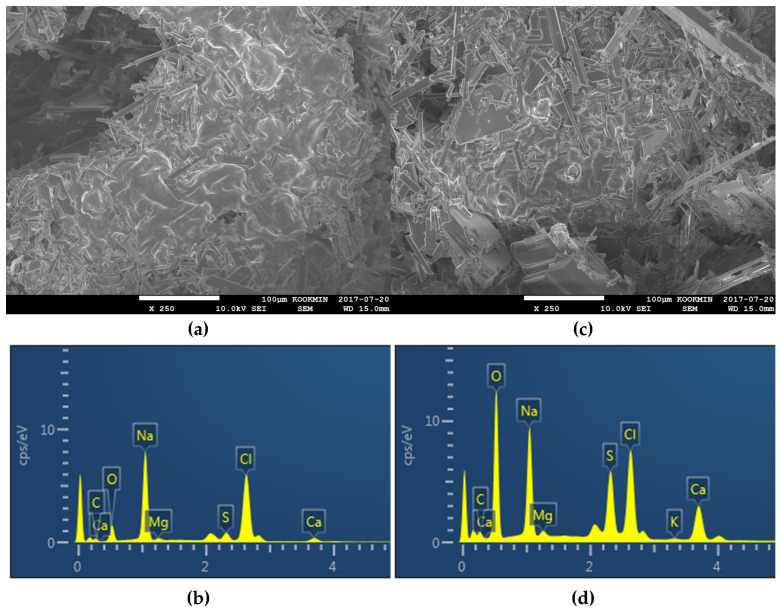
SEM/EDX analysis of fouling matters under (**a**,**b**) 60 °C and (**c**,**d**) 70 °C conditions by seawater.

**Figure 8:**
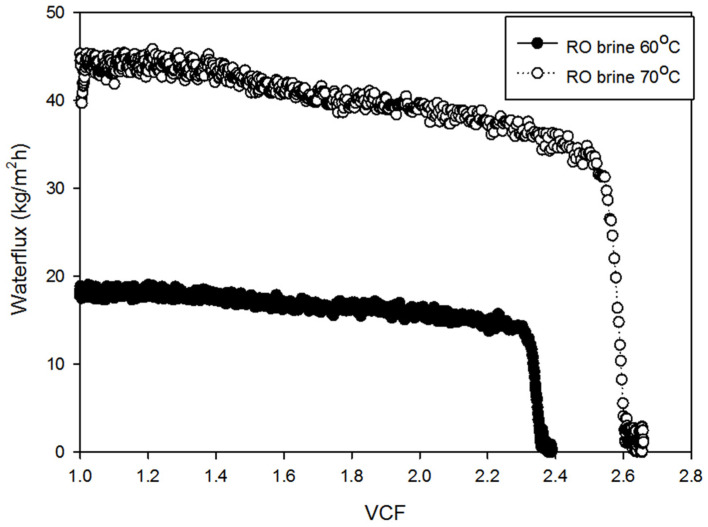
Waterflux by RO brine operation: 60 °C and 70 °C.

**Figure 9:**
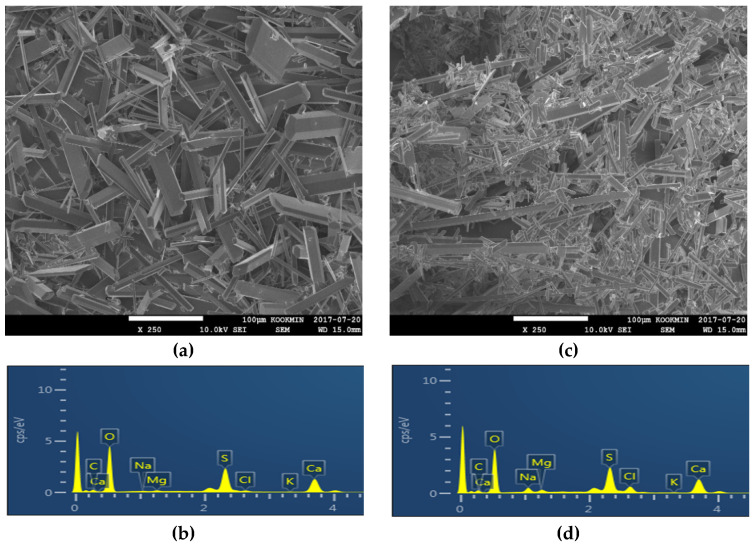
SEM/EDX analysis of fouling matters under (**a**,**b**) 60 °C and (**c**,**d**) 70 °C conditions by RO brine.

**Figure 10:**
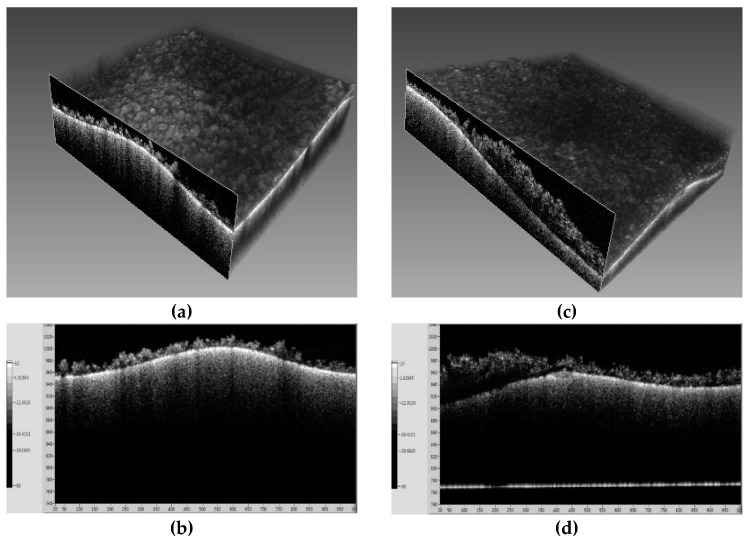
OCT images of membrane surface after (**a**,**b**) 60 °C and (**c**,**d**) 70 °C operation by RO brine.

**Figure 11:**
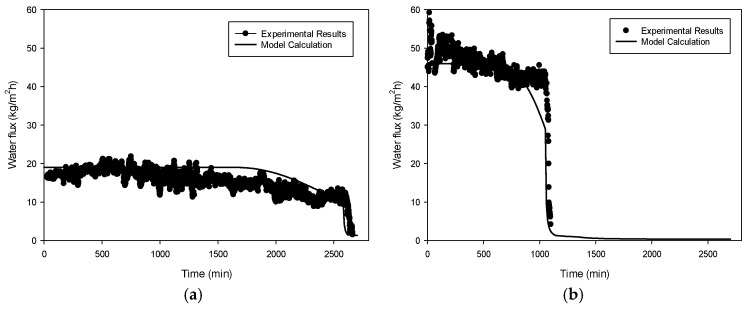
Comparison of experimental results with model calculation at feed temperatures of (**a**) 60 °C and (**b**) 70 °C. (Feed: seawater).

**Figure 12:**
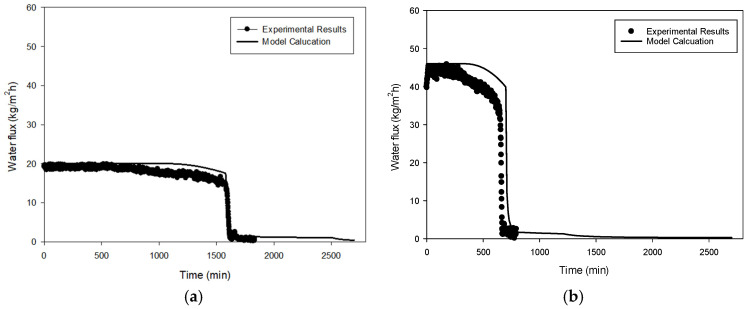
Comparison of experimental results with model calculation at feed temperatures of (**a**) 60 °C and (**b**) 70 °C. (Feed: SWRO brine).

**Figure 13:**
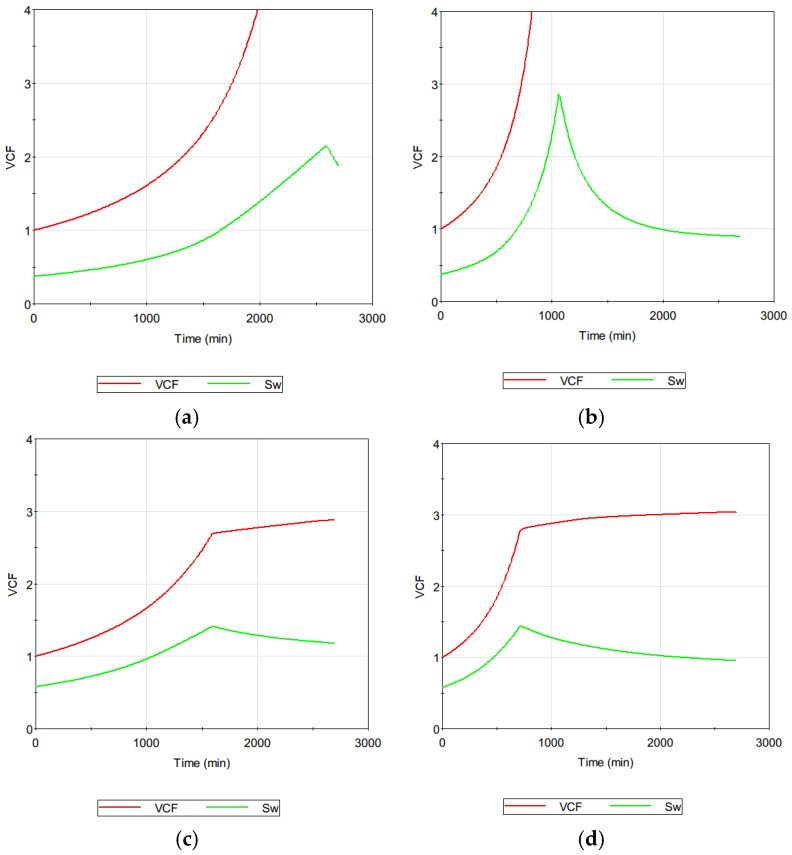
Analysis of VCF and SW for MD systems: (**a**) seawater at 60 °C; (**b**) seawater at 70 °C; (**c**) SWRO brine at 60 °C; (**d**) SWRO brine at 70 °C.

The experimental results for both seawater and RO brine under different temperature conditions are summarized in Figure 5, Figure 6, Figure 7, Figure 8, Figure 9, Figure 10, Figure 11, Figure 12 and Figure 13. The experiments were conducted at 60 °C and 70 °C using seawater (35,000 mg/L TDS) and RO brine (54,400 mg/L TDS) as feed waters to investigate membrane fouling. Each condition was tested using a VMD lab-scale system with a 1 L feed volume and a flat-sheet PVDF membrane having a membrane area of 12 cm^2^. Figure 5 shows the decline in water flux for seawater at 60 °C and 70 °C. At 60 °C, the initial flux was approximately 20 kg/m^2^h and remained relatively stable until around a VCF of 6.5. Beyond this point, a gradual decline was observed, with the flux decreasing steadily as the VCF increased, eventually reaching near zero around a VCF of 7.0. By contrast, the initial flux at 70 °C was significantly higher, around 50 kg/m^2^h, and remained stable until VCF 3.6. After this point, a sharp decline was observed, with the flux dropping rapidly and approaching near-zero levels by VCF 4. This earlier and more pronounced decline at 70 °C suggests that fouling was accelerated at the higher temperature, likely due to the faster crystallization of salts such as NaCl and CaSO_4_.

Figure 6 and Figure 7 present OCT and SEM-EDX images for the membranes fouled by seawater at 60 °C and 70 °C, respectively. Figure 6a shows a thin and uneven fouling layer at 60 °C, while Figure 6b reveals a thicker and denser fouling layer at 70 °C. SEM-EDX analysis, as shown in Figure 7a,b, confirmed that NaCl was the main fouling component at 60 °C, while Figure 7c,d at 70 °C shows a combination of NaCl and CaSO_4_, indicating that CaSO_4_ plays a significant role in high-temperature scaling.

Figure 8 depicts the flux decline patterns for RO brine at temperatures of 60 °C and 70 °C. At 60 °C, the flux initially remained stable around 18–20 kg/m^2^h until reaching an approximate VCF of 2.3. Beyond this point, the flux dropped sharply, approaching zero by a VCF of 2.4. By contrast, at 70 °C, the initial flux was significantly higher, around 40–45 kg/m^2^h. Although a gradual decline was observed with an increasing VCF, a steep drop occurred at an approximate VCF of 2.5, with the flux nearing zero by a VCF of 2.6. The faster and more pronounced flux reduction in RO brine compared to seawater suggests that the higher concentration of scaling compounds in RO brine leads to more rapid CaSO_4_ crystallization, resulting in more severe pore blocking. Figure 9 and Figure 10 provide OCT and SEM-EDX images of membranes fouled by RO brine. At 60 °C, Figure 9a shows a relatively thin fouling layer, while at 70 °C, Figure 9b shows a much thicker and denser fouling layer. The SEM-EDX results in Figure 10a,b confirm that NaCl was the dominant fouling component at 60 °C, while at 70 °C, both NaCl and CaSO_4_ were present (Figure 10c,d), contributing to the more severe fouling.

### 4.2. Comparison of Model Calculations with Experimental Results

The experimental data were used to verify and validate the model. There are four cases included in the data: (1) seawater (35,000 mg/L TDS) as the feed at 60 °C; (2) seawater as the feed at 70 °C; (3) SWRO brine (54,400 mg/L TDS) as the feed at 60 °C; and (4) SWRO brine as the feed at 70 °C.

In Figure 11 and Figure 12, the experimental flux (denoted as symbols) is shown as a function of time. Initially, the flux was approximately 18 kg/m^2^h. The flux remained constant until the elapsed time reached about 1800 min. After this time, the flux gradually decreased. Then, the flux abruptly dropped after 2600 min. These results suggest that there are two different scale formation mechanisms in this case, which makes the flux prediction difficult. Although the initial flux and the time of flux drop were different, similar trends can be found in the other conditions, as depicted in Figure 11b and Figure 12a,b.

Although the flux decline owing to scale formation is rather complicated, our system dynamics model exhibited a good match with the experimental flux. The overall flux tendency could be reproduced by the model as shown in Figure 11. The model slightly overestimated the flux in Figure 11a and Figure 12a,b and underestimated it in Figure 11b. Considering variations caused by random experimental errors during the flux measurement, It can be inferred that the model effectively replicates the trends observed in the experimental results.

### 4.3. Theoretical Analysis of Scale Formation in MD System

Since the model was validated with the experimental data, it was applied to obtain information on the hidden variables in the MD system. Figure 13 shows the VCF and S_w_ as a function of time for the previous cases. If there is no crystallization, the VCF and S_w_ should have the same patterns. However, as can be seen in Figure 13, they exhibited different results. This is attributed to the onset of crystallization above a certain S_w_. Once either surface crystal formation or bulk crystal formation occur, the CaSO_4_ concentration in the solution decreases, thereby affecting the S_w_.

When the seawater was used as the feed, the initial S_w_ was approximately 0.37. This is lower than the initial S_w_ for the SWRO brine, which was approximately 0.58. Since the initial S_w_ was lower for the seawater than the SWRO brine, the final S_w_ was expected to be also lower. However, the results were opposite as demonstrated in Figure 13. This is because the induction time for the SWRO brine is shorter than that for the seawater due to its higher CaSO_4_ concentration.

**Figure 14:**
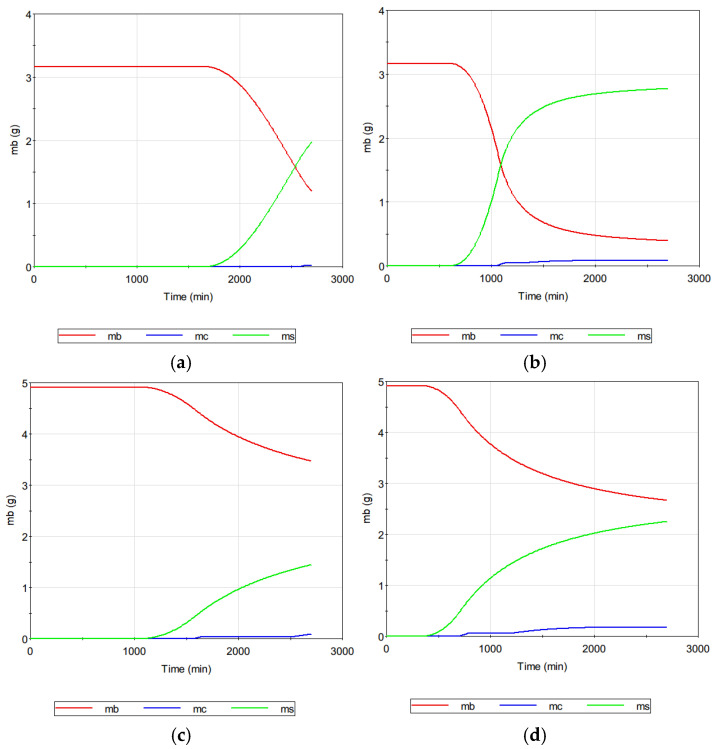
Analysis of solute mass (m_b_), surface crystal mass (m_s_), and cake crystal mass (m_c_) in MD systems: (**a**) seawater at 60 °C; (**b**) seawater at 70 °C; (**c**) SWRO brine at 60 °C; (**d**) SWRO brine at 70 °C.

Figure 14 compares the mass of dissolved CaSO_4_ ions in the retentate (m_b_), surface crystal mass (m_s_), and the cake crystal mass (m_c_) for the previous cases. Initially, all CaSO_4_ existed in the form of dissolved solutes. When the time exceeded a certain crystal value, the mass of the surface crystal increased. Subsequently, the mass of the cake crystals increased as well. The accelerated growth of the surface crystals can be attributed to concentration polarization, which elevates the c_w_ above c_b_. Despite this, it is clear that MD fouling caused by the development of cake layers from settled bulk crystals is also a considerable issue. This suggests that the control of MD fouling should be attempted based on a fundamental understanding of the scale formation mechanisms.

## 5. Conclusions

This research explored scale formation in MD systems by employing a system dynamics model built upon crystallization theory and the resistance-in-series model. Comprehensive experimental evaluations and advanced modeling led to several key findings:

1. The experimental results indicate that the flux decline in MD systems is controlled by two primary mechanisms: surface crystal formation on the membrane surface and bulk crystal formation in the solution phase. The initial decrease in flux is linked to surface crystal formation on the membrane, whereas the later and more significant flux drop is caused by bulk crystal formation occurring in the solution.
2. The system dynamics model was developed by incorporating complex interactions among variables and parameters. Despite inherent experimental variability and random errors, the model demonstrated strong predictive capabilities by accurately reproducing the observed trends in experimental flux decline. This suggests that the model is robust and reliable for predicting fouling behavior in MD systems.
3. One of the strengths of the model is its ability to provide detailed information on hidden variables such as the supersaturation ratio (S_w_), mass of dissolved solutes (m_b_), surface crystal mass (m_s_), and cake crystal mass (m_c_). These variables are challenging to measure directly in experimental settings but are crucial for understanding the underlying fouling mechanisms. Such insights are expected to guide the development of more effective fouling-control strategies for MD applications.

Overall, the combined use of experimental data and system dynamics modeling provides a comprehensive understanding of scale formation in MD systems. This work contributes to the ongoing efforts to mitigate fouling in MD by offering a validated model that can predict scaling trends under various operational conditions. Future research should focus on refining the model to include additional interactions among scaling components and applying these insights to optimize MD system performance.

## Figures and Tables

**Table 1:** Physicochemical characteristics of raw seawater and RO brine used in the experiment.

Parameter	Sea Water	RO Bine
pH	8.1	7.6
TDS (mg/L)	35,045	54,400
Chloride (mg/L)	20,069	32,600
Sulfate (mg/L)	2699	5050
Magnesium (mg/L)	1495	6100
Calcium (mg/L)	465	1760
Sodium (mg/L)	10,899	17,330

**Table 2:** Parameters and assumptions used in this study.

Items	Meanings	Values	References
*C*	Constant	90,700 m^6^ K^3^ (mJ)^−3^	[34]
*σ*	Surface energy of the crystal (in the absence of additives)	9.4 × 10^−3^ J/m^2^	[34]
*n*	Reaction order of surface crystal formation	1	[35]
*m*	Reaction order of bulk crystal formation	1	[35]

## Data Availability

The original contributions presented in the study are included in the article, further inquiries can be directed to the corresponding author.
